# Molecular taxonomy of nociceptors and pruriceptors

**DOI:** 10.1097/j.pain.0000000000002831

**Published:** 2023-01-25

**Authors:** Jussi Kupari, Patrik Ernfors

**Affiliations:** Division of Molecular Neurobiology, Department of Medical Biochemistry and Biophysics, Karolinska Institutet, Stockholm, Sweden

## 1. Introduction

The ability to feel the warmth of the sun or the coolness of a breeze on a summer's day; a pleasant caress of skin; to explore the texture, size, and shape of an object; and to feel pain as a mechanism to protect us from what is dangerous around us has fascinated mankind for centuries. Perhaps this fascination is rooted in the fact that the somatic senses of temperature, pain, touch, itch, and proprioception endow us with the ability to continuously stay in contact with the world within and around us. Attempting to explain how we react to heat, the 17th century philosopher René Descartes depicted a thread connecting the skin with the brain and particles of fire pulling on the thread.^20^ In the 1880s, distinct sensory spots on the skin were recognized and shown to respond to specific stimuli, such as touch, heat, or cold. This finding was taken as evidence for different sensations relying on the existence of distinct nerves tuned to specific types of stimuli.^83,94^ In the beginning of the 20th century, the discovery of different nerve fiber types with distinct conduction velocities, activation thresholds, and refractory periods made it possible to link specific fiber types to unique qualities of sensation, such as proprioception and different kinds of touch. Unlike innocuous touch and pressure, which are communicated through nerve fibers responding only to mechanical stimuli, noxious stimuli were found to activate a class of polymodal nociceptive nerve fibers responding to several kinds of unpleasant stimuli, including high-threshold mechanical force and intense heat.^7,12^ This work led to the general idea that the capacity to discriminate perceptual qualities arises from unique functions of a variety of sensory neurons involved in somatic sensation. This is possible because different neuron types are tuned to respond and transduce certain, but not all, kinds of stimuli, such as warm and cool temperatures, noxious heat, noxious cold, light punctate sensation, painful pressure, sharp objects, hair pull, chemical irritants, inflammatory substances, chemical and mechanical itch, and various kinds of touch and vibration.^29,38^

Apart from providing sensory awareness about our body and its surroundings, the somatosensory system is also essential for other functions we perform effortlessly and without much thought. For example, a continuous flow of information in a sensory–motor feedback loop involving several dozen muscles is necessary to coordinate movements when taking a sip from a glass of water or for the seemingly simple task of walking. Somatosensory neurons are also organized in discreet pathways with autonomic sympathetic and parasympathetic motor neurons driving reflexes involved in physiological homeostasis, such as thermoregulation through reflex control of sweat glands and selective vasoconstrictor systems in muscle and skin active when standing up and during hypothermia, respectively.^16,49,74^ Furthermore, pleasant and painful sensations involve not only discriminative perception and coordination of efferent reflex functions but also emotional and motivational components important for behavior. For example, social touch such as hugging and caressing induce sensations of pleasantness to facilitate emotional bonding, affiliative behavior, and well-being,^82,88^ while noxious stimuli not only convey sensory discriminative and protective reflexes but also include an affective-motivational dimension of unpleasantness contributing to long-term behavior avoiding harm.^112,125^

The existence of molecularly unique neuron types coding for each quality and dimension of somatosensation is unlikely. Instead, summation of activity and inactivity from different types of nerve fibers is likely to contribute to the sensory qualities and dimensions involved. Several recent studies support this conjecture, eg. the discovery of warmth sensation relying on the simultaneous activation of warmth-sensing and inhibition of cold-sensing nerves^97^; the requirement of different sensory neurons for protective reflexes and affective coping behavior elicited by the same stimuli^45^; the suppression of noxious cold (−10 to 10°C) by Calca^+^ neurons^18,81^; the modulation of touch-evoked itch by Merkel cells and *Slc17a8*-lineage (low-threshold mechanoreceptor [LTMR]) sensory neurons^32,33,108^; and alleviation of Npy2r^+^ neuron-dependent acute pain behavior by the simultaneous activity of the A-LTMR touch neurons.^3^ With a thorough molecular categorization of sensory neuron types, experiments can be designed with even greater precision, opening possibilities for gaining new, deeper insights into the cellular basis for nociception and pruriception.

## 2. Categorizing sensory neurons

Sensory neurons have historically been classified based on physiological properties, morphology, target innervation, spinal termination, developmental hierarchy, and functional and neurochemical properties (reviewed by Emery and Ernfors, 2020^29^). This work has been instrumental for deciphering how the peripheral sensory nervous system is organized. However, these strategies have measured a dozen or so quantitative features, such as response to cold, heat, and mechanical stimuli; conduction velocity; neuron size; or a handful of marker genes, which might not be sufficient for reliable identification of functional units. By contrast, single-cell RNA sequencing (scRNA-seq) can measure thousands of features, is quantitative, and allows for an unbiased classification of cell types. Nevertheless, the accuracy of cell-type assignment is affected by data quality (library complexity, sequencing depth, technical errors, and technical noise) and the fact that only a proportion of the messenger RNA (mRNA) molecules within a cell is successfully converted for sequencing. In addition, different cell types contain different quantities of mRNAs resulting in differences in sequencing quality, affecting cell-type assignment. This is particularly relevant for sensory neurons which, unlike most neurons of the central nervous system, are of highly variable size. Cell-type identification is also affected by the resolution used for clustering. The aim of clustering in scRNA-seq is to identify the diverse neuron types which represent unique functional units. However, all molecular cell-type taxonomies are hampered by the questions “what is the relation between variable molecular features and function?” and “what is a cell type?”^152^

Sensory neurons are intimately associated with a surrounding sheath of satellite glial cells which is often insufficiently removed during sample preparation. Single-nucleus RNA sequencing (snRNA-seq) can be used to mitigate this. Although most of a cell's genomic information can be accessed in the nucleus alone, there is less starting material when the cytosol is not included leading to a lower median number of unique genes detected per cell and the presence of ambient RNA confounding classification. Gene capture is a vital metric, and although the ease of clustering and identification of cell types might be improved in snRNA-seq through increasing the number of analyzed cells, much of biological insights into cell-type function can be lost due to the detection of a smaller proportion of the genes expressed by the cell. Because of this, we have always favored data generated by scRNA-seq and will in this review focus on results obtained by this method. Although there are several scRNA-seq studies published on the heterogeneity of mouse somatosensory dorsal root ganglion (DRG),^14,60-62,114,137,151^ trigeminal,^65,91,145^ and jugular^60^ neurons, there has been a general lack of efforts to integrate the data to find recurrent cell populations across the different studies using unbiased computational strategies. For this purpose, we have used intersample mapping to propagate information between studies and machine and deep-learning strategies to find neural correlates between our studies^60,61,131,151^ as well as the study of mouse DRG by Sharma et al.^114^ Because we know the relation between sequenced cells in these studies, our taxonomy of sensory neurons is heavily influenced by the molecular features as revealed in these studies.

## 3. Strategy of somatosensation along the body axis

General somatosensory neurons are present in the trigeminal ganglia, the superior and inferior (jugular) ganglia of the glossopharyngeal and vagal nerves, and the DRG, with a very small number in the geniculate ganglion.^19,133^ In addition, some accessory general somatosensory neurons may be present along the XI^th^ cranial nerve. Sensory neurons located in the trigeminal ganglia convey sensory information from the face and oral cavity (including the dura mater and teeth, respectively), lips and mouth having the greatest representation. Neurons from the geniculate and jugular ganglia innervate parts of the head, the ear, and throat while DRG neurons convey cutaneous and deep sensation from the rest of the body. Unlike somatosensory neurons which detect and relay conscious sensations, visceral sensory neurons are largely involved in reflex functions regulating body homeostasis related to organ function and transmitting gustatory information from taste buds.^60,79,136,140^ These neurons are organized in cranial ganglia, including the petrosal and nodose ganglia which are dedicated visceral ganglia. Comparing neuron types in the general somatosensory trigeminal, jugular, and DRG identified by scRNA-seq suggests that the neuronal basis for somatosensation is conserved along the body axis, while visceral sensory neurons of the nodose ganglion are represented by completely different kinds of neurons.^60^ However, scRNA-seq of colonic DRG neurons^43^ has led to the identification of neuron types not found in other scRNA-seq studies of the DRG, suggesting the existence of somatosensory neuron types dedicated to visceral innervation. The data from this study have not yet been compared with other DRG scRNA-seq data sets using unbiased computational strategies, and thus, the similarities and differences to neurons identified in other studies remain to be explored.

## 4. Overview of sensory neuron types and their putative functions

**Figure 1.**
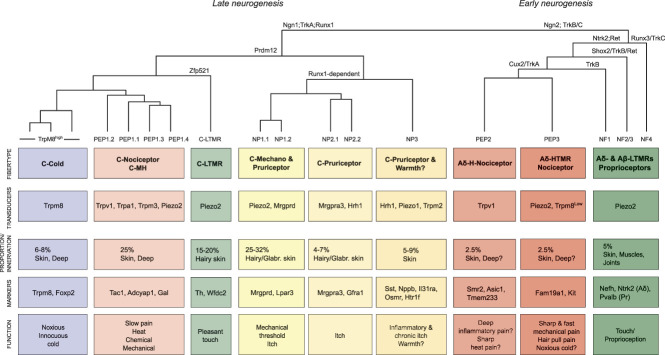
Molecular and functional taxonomy of mouse dorsal root ganglion sensory neuron types. Molecular types and hierarchical relationship are based on highly variable genes in mouse scRNA-seq data. The classification is based on Usoskin et al. (2015), Zeisel et al. (2018), and Sharma et al. (2020), and the nomenclature which follows Emery and Ernfors (2020) is indicated at the end branch of the hierarchical tree. The first or top box represents a conflation of hierarchically related neuron types with information summarizing fiber type (C, Aδ, and Aβ), modalities of sensation evidenced by functional experimentation or predicted based on expression (M, mechanical; H, heat; HTMR, high-threshold mechanoreceptor; LTMR, low-threshold mechanoreceptor). Boxes from top to bottom: neuron type, major sensory transduction genes expressed, percentage of representation among all DRG neurons, innervation targets, examples of marker genes, and finally function of the neurons. Question mark indicates prediction based on expression. Note that the hierarchical relationship largely reflect ontogeny which is outlined in the hierarchical tree as examples of transcription factors and receptors. The neurogenic transcription factor Neurogenin-2 (Ngn2) drives early neurogenesis generating Aδ nociceptor, Aβ LTMR, and proprioceptor neuron types which diversify through transcriptional activation, repression, and signaling through Trk and Ret receptors. Neurogenin-1 is responsible for neurogenesis of later born neurons defined by Runx1, Prdm12, and TrkA signaling which diversify into mostly unmyelinated nociceptive and pruriceptive neurons. Nppb, natriuretic peptide precursor B; scRNA-seq, single-cell RNA sequencing; Sst, somatostatin.

Based on the taxonomy presented here, 19 molecular types of DRG sensory neurons are predicted. An overview of the neuron types, expression of sensory transducers, percent contribution to neurons of the dorsal root ganglion, peripheral innervation, key markers, and function is outlined in Figure 1. This figure also shows the hierarchical relation between the different adult neuron types as well as key transcription factors and growth factor receptors known to drive cell-type diversification during development. Interestingly, the molecular relation in adult largely reflects ontogeny.

Neurons of the atlas include the innocuous mechanoreceptors represented by 1 type of Aδ-LTMR, Aβ rapidly adapting (RA) LTMR, Aβ slowly adapting (SA) LTMR, proprioceptor, and 1 C-LTMR, showing overall consistency with functional units explored in the mouse and human.^2,38,154^ Although this review is not focusing on LTMRs, it is interesting to note that 2 functional features seem to separate LTMRs into distinct molecular types: rate of adaptation and conduction velocity. The molecular properties underlying these features could be mutually exclusive and because of this, separate as unique entities defining LTMR types. By contrast, the molecular classification of A-LTMRs does not reflect peripheral termination morphology. For example, only 1 type of Aβ RA-LTMR neuron type has been identified by scRNA-sequencing,^114,131,151^ which terminates as longitudinal lanceolate endings in hair follicles and in the Meissner and Pacinian corpuscles.^38^ Furthermore, the fibers of 1 type of Aβ SA-LTMR terminate either in Merkel cells or putatively also as circumferential endings in hair follicles.

Machine learning–based analyses have revealed that Usoskin et al.,^131^ Zeisel et al.,^151^ and Sharma et al., 2021^114,131,151^ (from now we refer to these studies as only “Usoskin,” “Zeisel,” and “Sharma” for brevity) independently identified the same nociceptor and pruriceptor neuron types in mouse DRG, although with varying resolution. The low-resolution view reveals the major divisions of neuron types, whereas at high resolution the individual subtypes within the major divisions can be identified. At high resolution, there are 14 types of nociceptors and pruriceptors with predicted C-fiber or Aδ-fiber properties. These can be conflated into 7 major types with subtypes based on the hierarchical relationships. We believe the subtypes represent functionally similar units because many of the known transducers for somatic sensations such as Trpm8, Trpv1, Trpm2, Trpm3, Piezo2, and a multitude of itch receptors are consistent with both the conflated and high-resolution taxonomy. The conflated taxonomy of nociceptors and pruriceptors include the following neuron types using the Usoskin nomenclature: TrpM8^high^, PEP1-3, and NP1-3. The full taxonomy includes 3 types of cold-sensing “TrpM8” neurons, 4 types of mechano-heat polymodal nociceptors (peptidergic “PEP1” neurons), 2 Aδ-nociceptor neuron types (putative heat or inflammatory peptidergic “PEP2” and putative a high-threshold mechanoreceptor type (A-HTMR) peptidergic “PEP3” neurons), 2 Mrgprd^+^ neuron types involved in light punctate reflex withdrawal and β-alanine itch (nociceptor and pruriceptor 1 “NP1” neurons), 2 Mrgpra3 neuron types involved in itch (nociceptor and pruriceptor 2 “NP2” neurons), and 1 somatostatin (Sst) and natriuretic peptide precursor B (Nppb) expressing neuron type involved in itch, allergic itch, and possibly perception of warmth (nociceptor and pruriceptor 3 “NP3” neurons) (Fig. 1). Although most of these major types have been shown to represent distinct functional units, unique functions for some of the subtypes remain to be shown. For example, there are 4 PEP1 subtypes, yet unique functions as compared to the other 3 have only been demonstrated for one of these 4. Conversely, Usoskin identified 2 and both Zeisel and Sharma one molecular type of proprioceptive neurons,^114,131,151^ while recent studies focusing on proprioceptive neurons found greater molecular heterogeneity, corresponding to subtypes for Ia, Ib, and type II proprioceptive nerve endings.^95,144^ Furthermore, a unique neuron type expressing Mrgprb4 has been functionally described^135^ but is yet to be identified in scRNA-seq studies. It is likely to be embedded among the NP2 neurons^72^ and fails to separate as a distinct cell type due to its scarcity. Better resolution and discovery of additional molecular neuron types could be expected by increasing the quality and number of sequenced cells.

## 5. Cross-species strategies of somatosensation

DRG neurons from nonhuman primate (NHP, rhesus macaque) have been analyzed by scRNA-seq.^61^ Integration and co-clustering as well as machine learning comparisons between high-quality mouse and NHP scRNA-seq data sets have revealed the existence of corresponding cell types between species, suggesting that the basal layout of sensory neuron types is largely conserved between these species.^61^ Nevertheless, when looking at the level of individual genes, major differences in expression between the corresponding neuron types of the mouse and NHP are observed, some of which directly relate to transduction of somatosensory stimuli. Transcriptomic analysis of human DRG has been performed using spatial transcriptomics^128^ and snRNA-seq.^90^ There is a lack of validated computational strategies for comparisons between data obtained from scRNA-seq and spatial transcriptomics. Therefore, the molecular correlates between human neurons as identified by spatial transcriptomics and mouse or NHP neurons have not been extensively analyzed.^128^ Nguyen et al.^90^ identified 15 clusters based on snRNA-seq of human neurons (predicted neuron types, H1-H15) and identified mouse–human cell type counterparts based on some variably expressed genes and co-clustering. Although some clusters could not be mapped back to any mouse counterpart (H4 and H12 clusters), others showed resemblance to mouse (H1, H2, H5 clusters corresponded to mouse PEP1 C-polymodal subtypes; H8 to mouse TrpM8^high^ cold-sensing neurons; H9 to mouse PEP3; H10 to mouse NP1; H11 to mouse NP3; H13 to mouse Aδ-LTMRs; H14 to mouse Aβ-LTMRs; H15 to mouse proprioceptors; and C-LTMRs were not found), although some markers and inconsistencies were observed in the mouse–human comparison. These results give a first indication of the neural basis for human somatosensation; however, further studies are needed to understand the exact relationship and molecular makeup.

**Figure 2.**
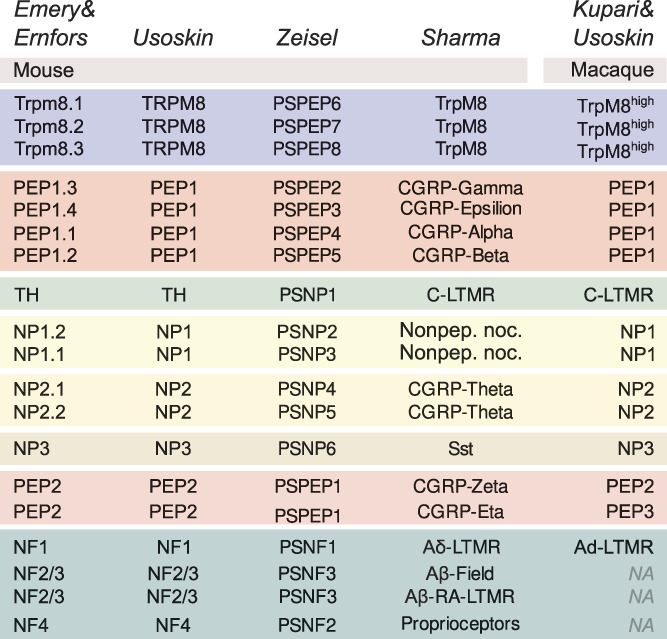
Schematic overview of the corresponding dorsal root ganglion neuron types between mouse and macaque data sets and “Usoskin,” “Zeisel,” “Sharma,” and “Kupari and Usoskin” nomenclatures. LTMR, low-threshold mechanoreceptor. NA, not available.

In mouse and NHP, the current data quality and molecular taxonomy of sensory neurons open for the assignment of function to defined neuron types with insights into the molecular features shaping their physiology. Below we describe the molecular heterogeneity of mouse and NHP sensory neuron types based on scRNA-seq studies where cell type correlates between different laboratories and studies have been shown using unbiased computational strategies^120,138,156^ and further summarize known functions of the different neuron types with a focus on nociceptive and pruriceptive neurons. The cell-type correlates and nomenclature used in these studies are presented in Figure 2. For those interested in interactive reading, we generated a single web site for the mouse Zeisel and Sharma data sets with an option to select the different cell-type nomenclatures (“Usoskin,” “Zeisel,” or “Sharma”) when querying gene expression in each of the different data sets (https://ernforsgroup.shinyapps.io/MouseDRGNeurons/) and a single web site containing both the STRT2 and SmartSeq2 macaque data (https://ernforsgroup.shinyapps.io/macaquedrg/). Expression of genes makes sense only when placed in known cell types. Thus, a molecular taxonomy of sensory neuron types is essential for insights into the neural basis of somatosensation and for understanding what goes wrong in chronic pain. Because of this, the review is centered around cell types rather than individual genes or molecules and there is a disproportionate impact by studies examining cell-type function by genetic strategies for ablation or by synthetic activation and inactivation of different neuron types using optogenetics or chemogenetics in mice. Furthermore, for those interested in LTMRs, we refer to other reviews on this topic.^2,3,45,70,87,94,112^

## 6. TrpM8^high^, C-cold-sensing neurons

Mouse TrpM8^high^ neuron type is named “high” because in macaque (PEP3, see below) and possibly also in human another neuron type also expresses Trpm8, although at much lower level. The TrpM8^high^ neuron type includes small-sized neurons critical for cool sensation that also importantly contribute to sensing painful cold. They innervate the skin and likely also mouth and esophagus,^56,150^ representing about 6% to 8% of all DRG neurons.^21^

This neuron type expresses unique features including Trpm8 and Foxp2 but lacks the mechanosensitive channel Piezo2 and heat-sensitive TRP channels (ie, Trpv1, Trpa1, Trpm2, and Trpm3). Usoskin and Sharma identified only one type of TrpM8^high^ neuron, while Zeisel identified 3 subtypes. This difference is due to the strategy and resolution used for clustering and identification of cell types.^61^

Based on marker expression associated with myelination, Zeisel predicted 2 subtypes representing putative C fibers and 1 putative Aδ fiber.^151^ Whether or not the 3 TrpM8^high^ subtypes distinguished by Zeisel represent functionally different neuronal types remains to be shown. Intriguingly, 3 functionally different subtypes of Trpm8^+^ neurons have been identified in the mouse trigeminal ganglion using Ca^2+^ imaging: 1 type that is active only during cool temperatures (28-20°C), another active only at noxious cold temperatures (<18°C), and a third with a hybrid response.^148^

Consistent with the unimodality of TrpM8^high^ cold-sensing neurons, genetic knockout studies have revealed the TRPM8 channel as a critical transducer for cool temperatures in the mouse.^4,17,22^ Mice lacking TRPM8 also display a clearly reduced aversive behavior toward noxious cold, indicating that the TRPM8 ion channel contributes to, but is not essential for, noxious cold sensation. By contrast, ablating the entire population of Trpm8^+^ sensory neurons leads to a complete loss of cold sensitivity and a greater loss of noxious cold sensitivity compared with mice where only the *Trpm8* gene has been knocked out.^57,87,101^ These studies highlight the significant role of TrpM8^high^ neurons in innocuous and noxious cold sensing, while simultaneously indicating that other neuron types may also participate in the detection of noxious cold through a mechanism independent of the TrpM8^high^ neuron types. This conclusion is also supported by another set of experiments. Low-threshold cold-sensitive fibers are largely unimodal consistent with the TrpM8^high^ neurons, while high-threshold cold nociceptors also respond to noxious heat and mechanical stimuli.^76^ Among C-fiber neurons, the TrpM8^high^ neurons are the only nociceptors not expressing Scn10a (Na_v_1.8). Consistently, deleting Na_v_1.8 in mice has no effect on cold behavior down to 5°C while responding to extreme cold stimuli (below freezing) is completely absent.^73^ Moreover, Calca^+^ (CGRP) and Scn10a^+^ C-fiber neurons of unknown molecular types were found to contribute to noxious cold sensing through a TRPM8-independent mechanism representing “silent” cold-sensing neurons sensitized during neuropathic cold allodynia.^75^ The identification of several channels proposed as alternative cold transducers^11,76^ has not helped in identifying neuron types contributing to the remaining noxious cold sensitivity in *Trpm8*-null mice, and furthermore, extreme cold below freezing seems to activate sensory neuron types regardless of type, likely because of tissue damage.^118^ Finally, TrpM8^high^ neuron types have been shown to also take part in cold-induced analgesia, indicating that activity in these neuron types can suppress pain.^103^

## 7. C-mechano-pruriceptors NP1, NP2, and NP3

The NP1, NP2, and NP3 classes include neurons involved in protective mechanosensory reflexes, itch, and potentially also nociception. In addition, these neurons express various thermosensitive TRP channels and, according to physiological recordings of cells or nerves, at least some are heat sensitive, although the in vivo role for noxious heat transduction in mice and humans seems negligible.^13,37,45,55,66,101^ Among these neuron types, NP3 is the most different in the mouse and the NHP, while the NP1 and NP2 types are hierarchically closer to each other.^61,151^ The thermosensitive and chemosensitive channels Trpv1 and Trpa1 are expressed in the NP-class neurons. This expression might reflect their role for signal transduction through receptors for histamine and nonhistamine itch-inducing compounds rather than contributing as heat sensors.^53,143^

Forced activation of the *Mrgprd*^Cre^ lineage neurons (including NP1, NP2, and NP3) by optogenetics fails to generate any place aversive behavior in naive mice, but does so in neuropathic animals.^139^ Thus, it has been proposed that none of the 3 neuronal types in the NP class are bona fide nociceptors, ie, high-threshold receptors with a function to transduce and encode noxious stimuli.^139^ Chemical itch is mediated through specific kinds of receptors whose expression patterns place chemical itch in the C-fiber NP class of neurons in the mouse and NHP. Exogenous and endogenous mediators released from damaged or infected tissues increase the extravasation of vessels attracting immune cells to the injured site for the inflammatory response.^77^ The released “inflammatory soup” is rich in purines, amines, cytokines, chemokines, lipid mediators, and growth factors. These include histamine, serotonin, leukotrienes, prostaglandins, bradykinin, and others of which some could directly activate or sensitize nociceptors and thereby mediate itch or pain. As is outlined below, NP2 and NP3 class neurons in mice are predicted to differentially respond to such mediators generating itch sensation while NP1 neurons are responsible for mechanical threshold detection and some types of itch. Consistent with this mouse taxonomy, itch in humans is conducted by nerves different from polymodal C-fiber nociceptors.^52,110^

## 8. NP1, C-mechanopruriceptor neurons

The nonpeptidergic NP1-type neurons are of small diameter, express Mrgprd in both mouse and NHP, and mostly innervate the superficial layer of the epidermis in the glabrous and hairy skin of the mouse.^156^ They are polymodal C fibers representing about 25% to 32% of DRG neurons and are involved in pruriception and threshold detection to light punctate mechanical stimuli (von Frey). This neuron type expresses unique molecular features, eg, Mrgprd, Mrgprx receptors, Lpar3, and Mdfic2. Zeisel defines 2 NP1 subtypes, while Usoskin and Sharma define only 1. This difference is due to the resolution used for clustering and identification of unique clusters.^61^ Whether or not the 2 subtypes of NP1 neurons represent functionally distinct units remains to be determined.

Based on physiological recordings, NP1-type neurons can be activated by mechanical, chemical, and heat stimuli in rodents^26,66,71,105^; however, although these neurons are critical for the withdrawal reflex evoked by punctate stimuli from von Frey filaments, their ablation does not affect heat or cold sensation.^13,45^ Furthermore, they are dispensable for the mechanical pain evoked by pinching.^44^ Consistent with the lack aversive behavior when activating all NP-class neurons (NP1-3),^139^ specific activation of NP1 fibers fails to generate any place aversive behavior in the naive animal,^6,139^ while it seems to do so after spared nerve injury and chronic inflammation (using complete Freund adjuvant).^1,139^ Thus, current studies support the idea that while NP1 neurons are not nociceptors in the naive state, they perform this role during chronic inflammatory pain. Still, the integrated contribution of NP1 neurons to mechanical allodynia during chronic pain, especially in relation to other neuron types, needs to be systematically evaluated. In the mouse, NP1 neurons are also involved in pruriception. Although NP1 neurons do not respond to histamine,^66^ β-alanine, a natural pruritogenic compound, directly binds and activates the MRGPRD receptor,^117^ which then engages TRPA1 and induces itch.^141^ Furthermore, the NP1 neuron type is the only one expressing the pruritogenic lysophosphatidic acid receptor Lpar3 in the mouse. Thus, in the naive situation, mouse NP1 neurons seem to primarily function as mechanopruriceptive neurons in reflex withdrawal to punctate stimuli and as sensors to some selected pruritogens. In NHPs, based on gene expression, NP1 neurons are predicted to have broader functions for itch, expressing receptors for histamine and different nonhistamine pruritogenic compounds, and often share expression with NP2 neurons, as will be described below.

## 9. NP2, C-pruriceptor neurons (chemo)

NP2-type neurons are of small diameter, express Mrgpra3 in the mouse, and mostly innervate the superficial layer of the epidermis while occasionally wrapping around hair follicles.^25,37,156^ They are polymodal high-threshold C fibers responding to both noxious mechanical and heat stimuli, but not cold,^37^ and represent 4% to 7% of sensory neurons.^25,155^ In vivo in the mouse, NP2 neurons have been proposed to be a neuron type dedicated to itch,^37^ although recent results suggest broader functions (see below). Zeisel defines 2 NP2 subtypes, while Usoskin and Sharma show only one.^114,131,151^ This difference is due to the resolution used for clustering and identification of unique clusters.^61^ Whether or not the 2 subtypes of NP2 neurons represent functionally distinct units remains to be determined.

Although the cells are heat sensitive at the cellular level, ablation of the Mrgpra3^+^ neurons in the mouse has no effect on pain behavior induced by heat and cold noxious stimuli, nor does it induce any shift in light punctate sensation evoked by von Frey hairs. Furthermore, no behavioral difference was observed in these mice in response to heat or mechanical stimuli in the inflamed animal.^37^ By contrast, mouse NP2 neurons are critical for chloroquine-induced itch mediated through the ligand activation of MRGPRA3 receptors that are uniquely expressed in these cells. Consistently, in mice where the Mrgpra3^+^ neurons are ablated, chloroquine itch is absent and histamine, serotonin, and several other itch agents display attenuated effects on induced scratching behavior.^37,104^ As in the case of MRGPRD, also MRGPRA3-dependent itch relies on TRP channels.^141^ Although NP1 and NP2 neuron types seem to be distinct functional units in the mouse, they seem to share many pruriceptive functions in NHPs. Contrary to the mouse, where chloroquine itch is mediated through the activation of MRGPRA3,^37,67,106^ this receptor is not conserved in humans, and chloroquine is instead engaging MRGPRX1.^67^ Thus, while chloroquine itch is conveyed by NP2 in the mouse, it is predicted to act in NHPs through both NP1 and NP2 neurons through the ortholog receptor MRGPRX1. A shared function of NP1 and NP2 neuron types in NHPs is predicted also for β-defensin-induced itch. β-defensin expression is elevated in the skin lesions of inflammatory dermal diseases such as psoriasis and atopic dermatitis, inducing itch through the activation of human MRGPRX1 and MRGPRX2 or mouse MRGPRA3 and MRGPRC11.^124,130,153^ While in the mouse β-defensins are predicted to act exclusively through the NP2 type of neurons based on receptor expression, in NHPs the ortholog receptors are expressed in both NP1 and NP2 neurons,^61^ again indicating species differences in the cell-type responses to external stimuli.

Itch can also be initiated through the protease-activated receptor F2RL1 (PAR2) by high levels of proteases observed in the skin of patients with chronic itch conditions, such as atopic dermatitis. In mice, PAR2 agonist-induced itch is absent in *Par2*-deficient mice.^5,116,123,142^ This receptor is expressed exclusively in NP3 neurons in mice where it is not only critical for itch but also evokes mechanical hyperalgesia.^40^ This functionality is not conserved in NHPs where PAR2 is not expressed in sensory neurons at all, indicating any contribution of PAR2 to itch in humans to be indirect, perhaps through skin cell types and/or immune cells.^50^ Although much focus has been on PAR2 as a contributor to itch in humans,^10,122,123^ the PAR family consists of 4 genes: *F2R (PAR1), F2RL1 (PAR2), F2RL2 (PAR3)*, and *F2RL3 (PAR4)*, which can be activated by endogenous and exogenous sources, including proteases. Nonhuman primate sensory neurons express PAR1 and PAR3 exclusively in the NP1, NP2, and the PEP2 type (an Aδ nociceptor). Thus, NP1 and NP2 neurons seem to be functionally similar in NHPs also in this respect. A shared function of NHP NP1 and NP2 neuron types is also predicted for cholestatic itch. Lysophosphatidic acid increases in sera of patients with cholestatic liver diseases and pruritus and can cause both pain and itch in humans.^27^ In mice, lysophosphatidic acid elicits both itch-related^39,59^ and acute pain-like behaviors, the latter being dependent on TRPV1.^48,93^ This is consistent with the effects of lysophosphatidic acid acting through LPAR3 expressed exclusively in mouse pruriceptive NP1 neurons and LPAR1 expressed in both pruriceptive NP3 and nociceptive PEP1 neurons (which also co-express TRPV1). By contrast, NHPs express LPAR3 in NP1 and NP2 neurons and LPAR1 in PEP2 (which also expresses TRPV1). Among the few pruritogens identified that mediate cholestatic itch apart from lysophosphatidic acid are bilirubin and bile acids^8^ which activate the MRGPRX4 receptor in human.^149^ MRGPRX4 is expressed in NP1 and NP2 neurons of NHPs. Thus, it seems that NP1 and NP2 neuron types are essential in NHP for chronic itch during cholestasis. Furthermore, a differential engagement of NP1, NP2, and PEP types of neurons by proteases and lysophosphatidic acid might represent itch and nociception, respectively, by the same compounds.

The proallergic cytokine thymic stromal lymphopoietin (TSLP) is a pruritogen^142^ acting through a heterodimeric receptor of IL7R and CRLF2 likely using an unknown indirect mechanism because sensory neurons do not express the receptor pair in either the mouse or NHPs. Another mast cell and keratinocyte released cytokine is IL33, which has been proposed to act directly on sensory neurons^64^ through the IL1RL1 (ST2) and IL1RAP receptors^78^; however, based on scRNA-seq, the effects are indirect as these receptors are absent in mouse and NHP sensory neurons.

Contrary to previous studies where activation of all NP class neurons (NP1-3) did not lead to aversive behavior,^139^ recent results indicate mouse NP2 neurons to be intrinsically multimodal based on their activation mode. Metabotropic activation evokes mostly pruriceptive behavior while ionotropic stimulation of the same NP2 cell type evokes pain-like behavior.^113^ This study corroborates NP2 neurons as pruriceptors but suggests that they can under some circumstances also function as nociceptors. Consistent with this, a putative contribution of NP2 neuron types to heritable musculoskeletal pain was inferred by partitioning genomic loci associated with chronic pain in human onto primate sensory neuron types. Genome-wide associations for pain converged on 2 different neuronal types, one of which was NP2.^61^ Thus, although a critical role of NP2 neurons for itch is firmly established, further studies on their contribution to chronic pain states are needed.

## 10. NP3, C-pruriceptor neurons (mechano-chemo)

The NP3 type neurons contribute to both mechanical and chemical pruriception and are predicted in mouse to also take part in warmth sensation. They are very small-diameter skin-innervating neurons^44^; express Sst, Nppb, and neurotensin (Nts); and represent about 5% to 9% of mouse dorsal root ganglion neurons in the adult.^131^

NP3 neurons are polymodal high-threshold C fibers responding to both noxious mechanical and heat but not cold stimuli,^44^ although the in vivo functional role for noxious mechanical and heat stimuli is likely negligible. Single-cell and bulk RNA-seq of mouse DRG have revealed expression of a rich repertoire of pruritogenic receptors in NP3 neurons, many of which are associated with inflammatory conditions and persistent itch.^120,131^ Thus, the unique expression of many of these receptors only in NP3 neurons predicts a central role for these neurons in responding to many pruritogens. Consistently, specific activation of NP3 neurons produces scratching behavior.^44^ It has also been shown that mice deficient in the neuropeptide NPPB, which is uniquely expressed in NP3 neurons, display a loss of behavioral responses to a range of pruritogenic agents (histamine, serotonin, PAR2 ligand, and chloroquine).^37,87^ Nonhistamine mast cell pruritogens, such as cysteinyl leukotrienes, interleukin-31, and sphingosine-1-phosphate,^5,42,120^ all produce itch and their cognate receptors are uniquely expressed in NP3 neurons (Cysltr2, Il31ra and Osmr, and S1pr1, respectively).^120,131^ These neurons also express TRPA1, which is required as a signaling component for itch.^42^ Serotonin^5,120^ can engage several receptors many of which are implicated in rodents.^7,65,97,100,125,133,133^ However, only Htr1a and Htr1f are robustly expressed in mouse sensory neurons and are specific for mouse NP3 neurons.^151^ Consistently, application of a serotonin receptor HTR1F agonist to cultured sensory neurons leads to calcium flux, but this effect is gone in cultures from mice in which the NP3 neurons were genetically ablated; furthermore, HTR1F agonist-induced itch behavior was reduced in such mice.^121^ Therefore, it seems that NP3 neurons are uniquely responsible for the direct itching effect of serotonin, cysteinyl leukotrienes, interleukin-31, and sphingosine 1-phosphate. Most of these functions are predicted to be conserved also in NHPs based on receptor expression. For example, S1pr1 is exclusively expressed in NHP NP3 neurons which also express the co-receptor Trpa1 and beyond this receptor, there are no other sphingosine 1-phosphate receptors in NHP sensory neurons. Nevertheless, some receptors for other pruritogens are expressed at lower levels also in NHP NP1 and NP2 neurons. For example, interleukin-31 receptors are expressed in NHP NP1 and NP2 in addition to NP3 neurons.^61^

As mentioned above, NP2 neurons contribute only partly to histamine-induced scratching in mice. It is possible that the remaining histamine itch phenotype is mediated through NP3 neurons because the histamine receptor Hrh1 is expressed in both NP2 and NP3 neurons in the mouse. By contrast, NHP NP3 neurons alone can be predicted to be critical for histamine-induced itch because HRH1 expression is abundantly present only in NP3 neurons. Itch induced by histamine is believed to rely on the downstream activation of TRPV1 by phospholipase-b3^47,115^ and both are expressed in the NP3 neurons of mouse and NHP. Thus, it seems histamine itch in NHPs relies on NP3 neurons while in the mouse, both NP2 and NP3 neurons could contribute.

Toll-like receptors (TLR2-5 and TLR7) have been implicated in itch by coupling to TRPA1 or TRPV1.^69,70,86,138^ Based on mouse scRNA-seq data, TLR4 expression is somewhat enriched in the PEP classes of neurons while TLR5 is present in LTMRs.^114,131,151^ However, NHP sensory neurons express robust levels of TLR2 in NP3 and A-LTMRs, TLR4 and TLR7 in PEP2 neurons, and consistent with mouse, TLR5 is expressed in A-LTMRs (MYD88 adaptor in all cell types). Thus, although a putative TLR5-dependent itch mechanism could be conserved across the species, it seems that NHPs have gained functions related to TLR2, TLR4, and TLR7 in sensory neurons.

Acute mechanical itch remains intact in animals after ablation of all sensory neurons expressing Scn10a (Na_v_1.8), which include all nociceptors and pruriceptors except the TrpM8^high^ neurons. Furthermore, activating Aβ-LTMRs through TLR5 with the bacterial protein flagellin—which in humans causes itch^92^—leads to predominantly TLR5-dependent scratching responses in mice.^96^ Both these results point to a role of Aβ-LTMRs for mechanical itch. However, recent results have linked mechanical itch to NP3 neurons.^41^ Hill et al.^41^ found largely reduced mechanical itch and hypersensitivity to mechanical itch (alloknesis) in mice lacking the mechanically activated ion channel PIEZO1. While PIEZO2 is a main known mechanosensitive channel in all other nociceptive and pruriceptive neurons, Piezo1 is uniquely expressed in NP3 neurons and seems to be dedicated for transducing mechanical stimuli into itch sensation. The authors speculate that a PIEZO1-dependent slow NP3 C-fiber itch mediates a persistent sensation and the desire to scratch to expel burrowing parasites from the skin, while Aβ-LTMR could trigger a rapid reflexive response.^41^ As mentioned above, engaging PAR2 using a selective PAR2 agonist leads to mechanical hyperalgesia.^40^ As Par2 is only expressed in NP3 neurons such mechanical sensitization could be caused by a change from PIEZO1-dependent itch to pain signaling by these neurons. In addition to pruritus, NP3 neurons might also contribute to the sensation of warmth and, perhaps through this, also affect pain sensitivity to heat. The warmth transducer Trpm2 is exclusively expressed in the NP3 neurons of mice^151^ and mice with a genetic deletion of Trpm2 display a robust deficit in the sensation of non-noxious warmth in the range of 33 to 38°C.^126,134^ However, warmth sensation in mice also requires Trpv1^+^ neurons^148^ (presumably PEP1 neurons), as well as Trpm8^+^ neurons (TrpM8^high^ neurons), with activity in the former neurons and inactivity in the latter.^97^ Thus, it is possible that warmth sensation in mice involves several neuron types, one of which is the NP3 type. Furthermore, elimination of the NP3-specific neuropeptide *Sst* using *Sst*^f/f^::*Trpv1*^Cre^ mice increases pain sensitivity to heat,^44^ suggesting a differential coding of sensory information carried by simultaneous activity of PEP1 and NP3 neurons, as opposed to only PEP1 neurons. This finding in mice might have a relation to psychophysical experiments in humans, where warming increases the threshold for heat detection without altering threshold responses of either heat-sensitive C or Aδ fibers.^100^ Another enigma is how NP3 neurons can initiate both thermal sensation and pruritus in mice. Based on gene expression, warmth sensation in NHPs involve different neural mechanisms because TRPM2 is not enriched in NHP NP3 neurons.^61^

## 11. PEP1, C-polymodal nociceptors

PEP1 neurons are small-diameter polymodal (mechanosensitive, thermosensitive, and chemosensitive) nociceptors that innervate the skin and deep tissues^98^ and represent about 25% of DRG neurons. In mice, these neurons express the heat-sensitive and chemosensitive transducers Trpv1, Trpa1, and Trpm3; the mechanotransducer Piezo2; the receptor tyrosine kinase Kit; and the neuropeptide substance P (*Tac1*).^132^ Usoskin^131^ identified one PEP1 neuron type while Zeisel and Sharma identified 4 subtypes.^114,151^ The increased resolution in Zeisel and Sharma is likely due to increased sample size in these studies.

The profound loss of heat and mechanical pain sensitivity in mice with ablation or chemogenetic inhibition of Na_v_1.8^+^ neurons (all but TrpM8^high^ and Aδ/β-LTMRs) has been largely ascribed to the PEP1 types. Furthermore, this neuron type is considered central for pain sensation because the C-fiber polymodal heat-sensitive and mechanosensitive neurons responsible for delayed and dull pain provide the basis for heat pain thresholds and contribute to hyperalgesia during injury in humans.^129^ However, unlike for several other neuronal types where unique markers have helped to unravel their contribution to function in the mouse, there are few studies restricted to studying only the PEP1 type of neurons. Common genes used to provide functional insights in the mouse include *Calca*^18,80^ and *Trpv1*^9,13^; yet, Calca is expressed in PEP1-3 and NP2 neurons and Trpv1 in PEP1, PEP2, and NP3 neurons with a lower level also in NP2 neurons. Nevertheless, as outlined below, PEP1 neurons are implicated as the main drivers for reflex-related and pain-related behavior to noxious heat, both acutely and during increased sensitization in inflammatory and neuropathic pain mouse models, as well as for pain-related coping (licking, shaking, and guarding) behavior connected to noxious cold and mechanical stimuli. Forced activation of Trpv1^+^ neurons (PEP1, PEP2, NP2, and NP3 neuron types) by optogenetics generates paw withdrawal, licking, and conditioned place aversion,^6^ demonstrating how these neurons contribute to pain behavior. Consistently, mice with ablation of both Trpv1^+^ and Trpm8^+^ neurons lack aversive responses to temperature in the range of 10 to 50°C.^101^ Similar results were obtained by ablating Calca^+^ cells (PEP1-3 and NP2 neuron types) or the central branches of Trpv1^+^ nociceptors, both of which selectively abolish reflex and coping behavior to noxious heat pain.^13,18,80^ In addition, coping behavior evoked by pinching is abolished in mice where Trpv1^+^ neurons have been silenced,^9^ while latency to respond is unaffected.^80^ A recent study finds that the ablation of Trpv1^+^ terminals in the adult mouse does not affect withdrawal responses to von Frey filament nor to noxious cold stimulation; however, there is a significant phenotype across all modalities (noxious cold, skin pinching, and hot plate stimulation paradigms) when measuring pain-related responses, such as coping behavior. Thus, they find that Trpv1^+^ nociceptors are required for both reflexes and coping behavior evoked by noxious heat, as well as for coping behavior elicited by cold and mechanical stimuli in the naive animal, suggesting that Trpv1^+^ nociceptors are required to drive acute and sustained pain.^45^ Therefore, reflex-related and coping-related behavior could be driven partly by different sensory neuron types with Trpv1^+^ neurons being critical for acute heat, mechanical, and cold pain-like behavior.^101^ This is consistent with the involvement of other neuron types such as NP1 for threshold detection and reflex behavior to punctate mechanical stimuli and TrpM8^high^ for cold threshold detection.

Beyond being detectors and transducers for heat, TRPV1 and TRPA1 are ligated by several different external and internal chemical irritants. Thus, they are polymodal receptors directly involved in inflammation and protection against chemical irritants. In models of neuropathic (spared nerve injury, SNI) or inflammatory (complete Freund adjuvant) pain, the situation is different from that of the naive animal. Silencing Calca^+^ neurons by optogenetics prevents spontaneous pain in injured mice (indicated by the place preference test), reverses both heat threshold hypersensitivity (beginning at ∼2 weeks after injury) and cold hypersensitivity (determined by withdrawal latency), and partially reverses sensitization to mechanical stimuli (allodynia measured by von Frey thresholds).^18^ Similarly, silencing Trpv1^+^ neurons abolish heat and mechanical inflammatory pain hypersensitivity, as determined by latency or force to withdrawal, as well as cold hyperalgesia.^9^ Brenneis et al.,^9^ also studied neuropathic pain but only 8 days after SNI when no heat hypersensitivity is observed. Nevertheless, the animals developed mechanical and cold allodynia and although silencing Trpv1^+^ neurons reversed mechanical hypersensitivity to baseline, cold allodynia was unaffected. Assuming the measured effect on behaviors is due to silencing the PEP1 neurons using the Calca and Trpv1 driver mouse lines, these studies indicate PEP1 neurons to be functionally polymodal and to represent the major neuron type responsible for slow persistent burning as well as dull pain during injury and inflammation. However, Aδ PEP2 neurons also express Calca and Trpv1 and thus, their contribution cannot be excluded (see below).

Temporal summation is documented for C-fiber-conveyed pain in humans. Here, increased firing by elevated intensity contributes to increased magnitude of pain with a near linear relationship between C-nociceptor impulses and pain rating.^129^ The unmasking of silent nociceptors during inflammation or neuropathy can also increase nociceptive input and contribute to hyperalgesia.^36^ Using microneurography, some unimodal C-fiber nociceptors (heat-sensitive or mechanosensitive) have been found to become responsive to heat and pressure when sensitized.^36,84,111^ These have been termed as silent nociceptors because they are normally unresponsive to naturalistic stimuli, but fire action potentials in response to electrical stimulation of the receptive field. Silent nociceptors are found mainly in deep tissues such as the knee joint and internal organs but also exist in the skin^30,34,46,109^ and can be sensitized after inflammation and neuropathy.^31,36,54,109^ Interestingly, one subtype of PEP1 neurons (named PSPEP2 in Zeisel, PEP1.3 in Emery and Ernfors 2020, and CGRP-gamma in Sharma) exclusively expresses Chrna3 in the mouse. Mouse Chrna3^+^ neurons were found to represent a silent nociceptor normally unresponsive to noxious mechanical stimuli, but which acquires mechanosensitivity when exposed to the inflammatory mediator nerve growth factor (NGF).^102^ These neurons seem to primarily innervate visceral organs and deep somatic tissues.^102^ Furthermore, a recent study found a set of sensory Prokr2^+^ neurons to innervate deep tissues, including hind limb fascia, and to represent the afferent component of the vagal anti-inflammatory reflex-attenuating systemic inflammation, such as during sepsis.^68^ Based on scRNA-seq, Prokr2 is expressed in the same neuron type as Chrna3.^151^ The conclusion that the PEP1.3 subtype represent neurons innervating viscera and deep tissues is consistent with the gene expression profile of these neurons. It is known from bulk RNA-seq analyses that markers enriched in sensory neurons projecting to deep tissues (muscle, bladder, colon, etc.) include Asic3, Prokr2, Chrna7, Serpinb1b, Smr2, and Fam19a1 (Tafa1),^43,147^ and some of these markers are enriched in the PEP1.3 subtype neurons. Thus, when combined, these results indicate that subtypes of PEP1 neurons identified by scRNA-seq represent unique functional units, and one these subtypes defines a deep tissue-innervating silent nociceptor that is recruited during chronic pain conditions, such as in neuropathy or inflammation.

The NHP correlate of PEP1 was identified using deep learning and other machine learning strategies; however, the sample size was too small to identify PEP1 subtypes.^61^ In similarity to mouse PEP1, NHP PEP1 expresses the heat-sensitive TRP channels TRPV1, TRPA1, and TRPM3^132^ and a number of conserved markers across the species such as TAC1, CALCA, ADCYAP1, GAL, NTRK1, and more.^61^ However, there is also important species-specific expression, eg, the sphingosine-1-phosphate receptor S1pr3 in the mouse and the purinergic receptor P2RY12 and the bradykinin receptor BDKRB2 in the NHP.^61^ Nevertheless, despite differences in expression of some transducers, analyses across species of the overall molecular features reveal no ambiguity regarding the relation between mouse and NHP PEP1, supporting the molecular relation across species.^61^

## 12. PEP2 and PEP3 Aδ nociceptors

Aδ nociceptors in the mouse are thinly to moderately myelinated fibers innervating the deep tissues and skin. These neuron types can be divided into 2 subtypes: a high-threshold heat-sensitive (A-H) type and A-HTMR, which together represent about 5% of DRG neurons in the mouse.^61,114^ While C-fiber pain is experienced as delayed dull and sometimes burning pain in humans, a heat-insensitive A-HTMR-type conducting at Aβ-fiber velocity evokes a sharp painful sensation of stinging and pricking when electrically stimulated.^12,89,129^ In addition, heat-sensitive A-fiber nociceptors exist in humans, although their contribution to heat pain is unclear.^24,85^ PEP2 and PEP3 are predicted to be lightly myelinated in the mouse and NHP, as inferred by NEFH and Cntnap2 expression.^61^ In the Zeisel and Usoskin data sets,^131,151^ mouse PEP2 and PEP3 were merged into 1 cluster named PEP2, while the increased resolution in the Sharma data led to the separation of these cell types in the mouse to PEP2 and PEP3 (originally named CGRP-zeta and CGRP-eta, respectively).^114^ In NHP scRNA-seq of DRG neurons, PEP2 and PEP3 were also identified as distinct cell types and found to correspond to PEP2 and PEP3 in Sharma.^61^

PEP2 could represent a lightly myelinated Aδ-heat neuron type based on the absence of Piezo2 and Trpa1 and the presence of Trpv1. Chrna7 is expressed in neurons innervating deep tissues and is selectively enriched in NHP PEP2. In many other respects, PEP2 share features with polymodal C-fiber nociceptors (PEP1), eg, in expression of Calca, Trpv1, Scn9a (Na_v_1.7), and Scn10a (Na_v_1.8), but unlike PEP1 do not express Scn11a (Na_v_1.9). In addition to a contribution of polymodal C-fiber nociceptors to heat pain,^24,85^ cutaneous first or fast heat pain has also been described in humans.^15,28^ Although it is unclear whether these are mechanically insensitive or not, PEP2 is the only A-fiber neuron type expressing a heat-sensitive ion channel, ie, Trpv1. However, TRPV1 is a polymodal channel not only activated by heat but also by low pH and numerous molecules associated with inflammation and tissue damage.^146^ Because of this, and molecular features of deep tissue-innervating neurons, it is possible that this neuron type is involved in first cutaneous heat pain and deep inflammatory pain, likely together with the more persistent slow pain elicited by PEP1 subtype of neurons.

PEP3 is predicted to be a lightly myelinated Aδ-high-threshold mechanoreceptor (Aδ-HTMR).^61^ The most notable differences between PEP2 and PEP3 are the marked enrichment of the mechanosensitive channel PIEZO2 in PEP3 and the absence of TRPV1 and TRPA1 in the same cell type. Thus, the PEP3 neuron type is predicted to be mechanosensitive, but heat and chemo insensitive. In addition, PEP3 expresses the cold transducer TRPM8 in NHP, albeit at a much lower level than the TrpM8^high^ type. PEP3 expresses markers for lightly myelinated neurons and, based on TRPM8 expression, might also conduct first or fast noxious cold sensation that can be experienced as pricking. This assumption is consistent with heat-insensitive mechano-cold-sensitive Aδ-fiber nociceptors signaling pain in response to extreme cold in the rat.^24,119^ In the mouse, the Calca^+^ but Trpv1^−^ neurons described by Ghitani et al.^35^ correspond to PEP3 neurons. These presumed PEP3 neurons were found to be Aδ-HTMRs which terminate as circumferential endings wrapping the base of the hair follicles and become activated by the pulling of a hair, possibly allowing accurate and rapid localization of mechanical pain.^35^ Another study used the *Npy2r* gene to study sensory neurons locating it mostly in Calca^+^ but Trpv1^−^ neurons innervating glabrous and hairy skin as free nerve endings.^3^ The only neuron type containing Npy2r and Calca but not Trpv1 in the taxonomy is the PEP3 neuron. The Npy2r^+^ neurons displayed Aδ-fiber conduction velocity were heat insensitive and, based on ablation experiments, play a critical role in initiating fast protective limb withdrawal to a sharp (dissecting) pin, while displaying unaffected latency of withdrawal to blunt punctate (von Frey) stimuli of the same force. Consistent with a nociceptor, optogenetic activation led to robust pain-like behavior.^3^ Activation of nociceptors with Aβ-like conduction velocity in humans (A-HTMRs) also leads to sharp, pricking pain with an exact location, suggesting a faster conduction in humans than in mouse.^89^

As noted above, based on the expression of low levels of TRPM8 in NHPs,^61^ the PEP3 neuron type could represent a hitherto unknown type contributing to acute noxious cold. Furthermore, a study has described cold-insensitive, Calca^+^ neurons becoming cold-sensitive in an animal model of neuropathic pain which could contribute to cold hyperalgesia.^75^ It remains to be shown if silent cold nociceptors are represented by PEP3 neurons.

## 13. NF1 Aδ-low-threshold mechanoreceptor neurons

Aδ-LTMRs (NF1 in the Usoskin nomenclature) represent the most sensitive of all touch receptors and are involved in sensing hair deflection and very fine intermittent tactile sensation.^107^ They are lightly myelinated, represent a few percent of DRG neurons, and terminate as lanceolate endings around hair follicles. NF1 neurons in the mouse are the only LTMRs expressing high levels of Ntrk2 yet lacking Ret.^63,107^ In addition, NF1 neurons express intermediate levels of Nefh,^131^ consistent with being thinly myelinated and having Aδ-fiber conduction velocity in the mouse.

Sensory testing has shown that capsaicin-induced mechanical hyperalgesia to brush-evoked pain relies on Aβ-LTMRs in humans because such pain is prevented after a nerve block of large A fibers.^51,58^ Studies in mice have revealed that during ongoing pain signaling in situations of neuropathy or during simultaneous firing of C-fiber nociceptors, Aδ-LTMRs seem to access spinal nociceptive pathways and encode pain instead of touch. In doing so, they have been proposed to contribute to mechanical allodynia.^23,99^ In rats, a general optogenetic activation of most or all innocuous mechanoreceptive A fibers after peripheral nerve injury also engages superficial dorsal horn neurons and elicits pain-like behaviors and aversion.^127^ However, the overall importance of Aδ**-**LTMRs for sensitization and chronic pain remains to be elucidated.

## 14. Conclusion

Establishment of a transcriptional taxonomy of somatosensory neurons using scRNA-seq has led to their unambiguous categorization in mouse and NHP based on molecular features. This has opened for the organization and partial reinterpretation of decades of research using gene expression markers, sometimes with previously unknown relation to actual neuron types. The emerging picture largely indicates that neuron types are tuned to respond preferentially to cold, heat, mechanical, or chemical itch and to convey slow or fast or sharp pain. However, a more complex image also emerges, whereby many qualities of sensation, such as for noxious cold, innocuous warmth, chemical itch, and mechanical and heat pain, rely on the integrated activity of several kinds of sensory neurons and where different behaviors, such as threshold reflexes and pain percept to the same stimulus are sometimes elicited by different neuron types. Moreover, sensory plasticity associated with hypersensitivity also involves functional changes in the response profile within the sensory neuron types. Thus, the limits of normal functions of sensory neurons seem not to be respected in ongoing and chronic pain. This transcriptional taxonomy of sensory neurons has already filled some knowledge gaps, and future experiments based on the taxonomy are expected to further clarify how different sensations are encoded and how molecular changes within neuron types and functional changes in the use of different sensory neurons contribute to chronic pain.

## Conflict of interest statement

The authors have no conflict of interest to declare.
